# Is non-conveyance solo-ambulances a useful mean to meet the increasing demand for emergency medical services in Denmark?

**DOI:** 10.1186/s12913-025-12448-8

**Published:** 2025-02-25

**Authors:** Frederikke Amalie Møller, Mads Lillethorup Persson, Elisabeth Larsen Engholm, Penille Horsbøl Kirkegaard Jensen, Ulla Vaeggemose, Tine Bennedsen Gehrt

**Affiliations:** 1 Department of Research and Development, Prehospital Emergency Medical Services, Central Denmark Region, Brendstrupgårdsvej 7, 2. th., Aarhus N, 8200 Denmark; 2 Department of Psychology and Behavioral Sciences, Aarhus University, Aarhus, Denmark; 3 Department of Clinical Medicine, Aarhus University, Aarhus, Denmark

**Keywords:** Emergency medical services [MeSH], Non-conveyance, Community paramedicine, Paramedics, Single responder, Employee perspectives

## Abstract

**Background:**

The growing demand for acute medical assistance creates an increasing pressure on emergency medical services (EMS) and emergency departments. This calls for initiatives to prevent avoidable admissions. A novel non-conveyance solo-ambulance was introduced in the Central Denmark Region; the Prehospital Visitation Unit (PVU). We investigated patient characteristics and prehospital management by the PVU, while exploring employee perspectives on the implementation process and how they perceived their expanded role and responsibilities working with the PVU.

**Methods:**

This mixed-methods study had a convergent parallel design. Patient data was collected on all patients assessed by the PVU between April 1st 2022 and April 1st 2023. Furthermore, 19 semi-structured interviews with paramedics, EMS dispatchers and technical dispatchers partaking in the operation of the PVU were conducted. Interviews were analyzed using thematic analysis with an inductive approach, drawing on principles from grounded theory.

**Results:**

Throughout the study period, the PVU served 1510 patients (median age: 60, IQR: 33–77). Among these, 83.6% were assigned an urgency level B, indicating acute, but not life-threatening, situations. Patients presented with a broad range of complaints, including a high number of patients with non-specific complaints. Paramedics completed treatment on-scene for 29.1% of all patients, spending a median time of 49 min (IQR: 33–64) on-scene. In the interviews, four themes were identified: (1) The implementation strategy had gaps, but was supported by ongoing adjustments, (2) Facilitating a patient-centered approach for the benefit of the patient and the system, (3) Community partnership and internal collaboration enabled paramedics as healthcare facilitators, and (4) Flexible workflows were needed to maintain professional agency.

**Conclusions:**

The PVU seems to offer a valuable alternative within the EMS, particularly for patients with non-specific complaints and conditions manageable at a lower level of care. Strong collaboration allowed paramedics to take on a facilitating role, creating appropriate pathways and providing patient-centered care. However, for successful implementation, ongoing adjustments were required, particularly in maintaining the professional autonomy of the healthcare professionals. As prehospital EMS increasingly respond to non-acute medical needs, initiatives like the PVU can play an important role in meeting growing demands.

**Supplementary Information:**

The online version contains supplementary material available at 10.1186/s12913-025-12448-8.

## Introduction

During the past decades, increasing demand for acute medical assistance has placed substantial pressure on prehospital emergency medical services (EMS) and emergency departments across European countries [1–5]. This trend seems to continue, as populations are ageing and the number of adults with multiple chronic conditions increases [5, 6]. Furthermore, a growing number of patients with non-urgent medical needs are assessed and treated within the prehospital EMS, creating a further strain on resources [7]. The increasing demand for prehospital EMS calls for new initiatives to reduce unnecessary ambulance use. Consequently, patients are increasingly treated and released on scene by EMS personnel, when transportation to the hospital is deemed unnecessary. Across European settings, 12–51% of all EMS assignments end in non-conveyance, the wide range possibly reflecting different definitions and possibilities of non-conveyance [8–11]. Internationally, these numbers vary even more from 3.7 to 93.7% [10].

Several countries across Western Europe (e.g. the Netherlands, Sweden, Finland, Denmark and Ireland) have recently implemented novel non-conveyance vehicles dispatched to low-urgency EMS calls, aiming to provide on scene treatment and referring patients to the appropriate level of care [12–16], thereby avoiding unnecessary transportation to the emergency department [13] and reducing patient waiting times [17]. In contrast to ambulances typically staffed with *two* healthcare professionals, these vehicles are mostly *single* responder units operated by one specially trained ambulance clinician, e.g. ambulance nurse, paramedic, or physician assistant [12, 15, 16].

These new initiatives have shown great potential with high levels of patient satisfaction [12, 16], reduced waiting times [17], cost-effectiveness [18] and maintained patient-safety [15]. However, to our knowledge, no studies have investigated the perspectives of the healthcare professionals operating and dispatching these vehicles. Understanding such perspectives are critical to support the implementation of new non-conveyance initiatives, as ambulance clinicians take on expanded roles and responsibilities.

The present study took place in the Central Denmark Region, Denmark, where a non-conveyance solo-ambulance operated by a single paramedic was introduced in March 2022; the Prehospital Visitation Unit (PVU). This study aimed to examine: (1) What were the characteristics of patients assessed by the PVU during the first year of operation? (2) How were patients managed within the EMS, when triaged to the PVU? (3) How did the healthcare professionals partaking in the dispatch and operation perceive their expanded role and responsibilities working with the PVU as well as the implementation process?

## Methods

### Study design

This mixed-methods study had a convergent parallel design, collecting quantitative and qualitative data during the same time frame [19–21]. The purpose of this design was to analyze the two datasets independently, but to integrate the results of the quantitative and qualitative analysis in the interpretation of the findings [21]. With this approach, we aimed to gain a deeper understanding of the patient care provided by the PVU that none of the data sources could have provided standing alone. We combined descriptive statistics of patients assessed by the PVU during the first year of operation, and 19 semi-structured interviews with paramedics, EMS dispatchers and technical dispatchers partaking in the operation of the PVU. Interview data were collected to contextualize and provide in-dept perspectives on the patient care provided by the PVU, as contextual factors are important in the implementation of new initiatives [22]. Likewise, patient data were included to underpin findings from the qualitative analysis, e.g. how patients were managed within the prehospital EMS. Results are presented using a contiguous approach [21], then integrated by relating findings from one data source to the other, when interpreting the results, focusing on relationships between findings from different data sources. The reporting of this study follows the Good Reporting of A Mixed Methods Study’ (GRAMMS) checklist (Additional File 4).

### Setting

The Central Denmark Region is one of five regions in Denmark, covering an area of 13,000 square kilometers, with a population of approximately 1,3 million inhabitants. All emergency calls are made through the national emergency number (1-1-2) and are initially answered by the police. In cases of medical emergencies, the call is forwarded to the regional Emergency Medical Coordination Center (EMCC), where assessment of patients is carried out by EMS dispatchers, who are healthcare professionals, e.g., nurses and paramedics [23]. Patient assessment is guided by the national criteria-based decision tool, Danish Index for Emergency Care, which divides patients’ complaints into 37 categories based on their main presenting problem [24]. Furthermore, this tool supports the EMS dispatchers in deciding an appropriate response based on the level of urgency (A-E; see Table 1) [24]. Technical dispatchers coordinate and ensure efficient dispatch of EMS vehicles (e.g., ambulances), monitor vehicle statuses, and optimize routes to provide timely assistance [23]. A physician-staffed mobile emergency care unit (MECU) can be dispatched in conjunction with the ambulance and the PVU. This physician can be contacted when paramedics make the decision to discharge patients on scene. Furthermore, a physician is available in the EMCC for conference.

**Table 1:** Urgency levels in the Danish index for emergency care [23, 25]

Urgency level	Description	Response
A - Acute	Acute situation assessed as potentially life-threatening	Immediate response during emergency call
B - Urgent	Urgent situation, but not assessed as acute life threatening	Dispatched within 30 min. of emergency call
C - Scheduled	Non-acute situations, but with need for observation and treatment in ambulance	Dispatched within 1–2 h of emergency call
D - Supine transport	Transportation while lying down, without need for observation or treatment	Planned response
E - Other services	Other help such as taxi, directing to other healthcare services, advice, etc.	No response

### The prehospital visitation unit (PVU)

The PVU was introduced in the Central Denmark Region in March 2022 as an addition to the current prehospital EMS set-up. The aim of this initiative was to prevent unnecessary admission to hospitals and enhance assessment of patients, including referral to other means of care (e.g. social services) or transportation (e.g. self-transport or supine transportation), as appropriate. Hence, the PVU could not convey the patient to the hospital. Patients assigned urgency level B were the primary target group for the PVU with the aim of targeting subacute patients possibly eligible for non-conveyance. However, later in the implementation it was decided to deploy the PVU to some assignment with urgency level A, when the PVU had a significantly shorter response interval compared to the closest ambulance or physician-staffed mobile emergency care unit. The PVU was operated by one paramedic and carried additional point-of-care testing (POCT) equipment for assessment of patients (e.g. measurements of CRP, lactate, and electrolytes) as well as a list of relevant telephone numbers for collaborating partners, such as shelters and other support services. These additional possibilities, in comparison to the ambulances, could support the paramedics with on-scene patient assessment and finding alternative care pathways within and beyond the healthcare system. Additionally, the PVU had all the standard possibilities for testing and treatment that the ambulances had (e.g. defibrillator, oxygen bag, vascular access kit). Paramedics recruited for the PVU underwent extra training days with a focus on subacute patient care. Throughout the study period, the PVU operated seven days a week from 7 am to 7 pm and covered primarily the largest city in the Central Denmark Region (Aarhus) with approximately 300.000 inhabitants.

### Data collection

#### Quantitative data

Descriptive data on all PVU assignments from April 1st, 2022, to April 1st, 2023, were included for data analysis, including assignments dispatched to pediatric patients. This included demographic data (age and sex), patient data (urgency level and main presenting problem), and prehospital management (time to arrival and how treatment was completed, e.g. patient handed over to other prehospital unit, patient refused transport, treatment completed on scene). All variables are routinely collected in the electronic prehospital medical record by paramedics and linked to the patient’s unique personal identification number [23]. To ensure workflows were well-established before data collection, data collected during the first month of operation were excluded from data analysis.

#### Qualitative data

Throughout its first year of operation, 19 semi-structured, in-depth interviews were carried out with employees deploying and operating the PVU; six paramedics, seven technical dispatchers, and six EMS dispatchers, who were purposely selected based on their experience working with the PVU, and to ensure variation on age, sex, and seniority [26]. All paramedics recruited for the PVU were highly experienced with a minimum of 5 years of experience within the EMS system. Given that the PVU operated with just one vehicle and only during daytime hours, the population with relevant experience working with the PVU was relatively small. Therefore, the majority of employees partaking in the dispatch and operation of the PVU were interviewed for the present study, e.g. we interviewed 6 out of 7 paramedics operating the PVU during the data collection period. By interviewing the majority of the professionals working with the PVU during the data collection period, we believe that that the interviews provide a reasonable representation of the perspectives of the population working with the PVU. This is further supported by the fact that all identified themes were present to some degree in every interview.

To gain knowledge on employee experiences throughout the first year of operation, interviews were carried out at three different time points: May 2022, February 2023, and March 2023. All interviews took place during working hours, occasionally interrupted by the professionals attending to patients. The interview guide was designed based on prior participant observation at the EMCC, providing insights into the workflow and terminology used by the employees, thereby creating an interview guide that resonated with the employees’ everyday experiences. Separate interview guides were developed for each of the professions interviewed to match their experience with the PVU (see Additional Files 1–3). Interviews were conducted by the third and fourth authors (ELE and PHKJ), audio recorded, and subsequently transcribed verbatim. Both interviewers had no prior relationship with the participants. All interviews were carried out and analyzed in Danish, and explanatory quotes and themes were subsequently translated into English by the research group.

### Data analysis

Descriptive data are presented as numbers (n) and percentages (%) or median and interquartile range (IQR), as appropriate. All statistical analyses were carried out in StataSE, version 18.0 (STATA Corp, College Station, Texas). The qualitative data was coded by the second author (MLP), who did not originally conduct the interviews, using thematic analysis and an inductive approach [25]. This approach offered significant advantages, as the researcher had no prior knowledge of the interview content or participants. This facilitated a genuinely inductive approach, minimizing bias and ensuring that the themes emerged from the data. This was considered the most appropriate approach, given the novelty of the PVU and sparse literature within the field, as it allowed us to uncover unexpected perspectives from the data. The coding process began by reading the interviews in their entirety with initial codes being noted. Afterwards, they were coded thoroughly with line-by-line coding of the transcripts, followed by grouping similar codes into broader categories. Themes were refined iteratively through discussions within the research team to ensure they captured the data’s key patterns and nuances [27].

During the initial coding, patterns and potential themes were identified within each of the professions (paramedics, EMS dispatchers, technical dispatchers). These potential themes were repeatedly compared to the body of data, both within and between professions, after which they were synthesized into four common themes that depicted how the professionals experienced operating and dispatching the newly implemented PVU. The analysis incorporated elements of grounded theory, mainly constant comparative methods, by systematically comparing emerging codes across all interviews. This approach allowed us to identify consistencies and divergences, enabling ongoing refinement of themes throughout the process [28].

The first author (FAM) coded a random sample of the transcripts (≈ 30%) to validate the themes developed by the second author (MLP), and to ensure thematic saturation by looking for any new themes emerging from the data. During this process, no new themes were identified. Moreover, it was important to ensure that the themes broadly represented the experiences across all interviews. To investigate this, we compared the frequency and context of citations coded to each theme across interviews and professions. All themes were present in some form in every interview, underscoring their relevance across the dataset, though themes varied in frequency and sentiment across interviews and professions, reflecting differences due to individual experiences and professional roles [29]. 

## Results

Throughout its first year of operation, the PVU was dispatched to 1.510 patients. 46.4% of patients were male and 41.8% were female, as 11.8% had missing data on age and sex due to no registration of their personal identification number. The PVU attended patients across all age-groups, though patients aged 65–79 years were the most common patient group, comprising 20.9% of patients attended. 1.263 assignments (83.6%) were deployed with urgency level B, indicating urgent, but not life-threatening, situations. However, the PVU also assisted in 132 assignments (8.7%) with the highest level of urgency (A). See Table 2. Patients assessed by the PVU were diverse in terms of their main presenting problem (Table 3). Most commonly, patients presented with impaired consciousness, paralysis, and dizziness (16.2%), unclear problems (14.3%), breathing problems (11.6%), abdominal or back pain (10.4%), and accidents (9.9%). The PVU spent a median time of 49 (IQR: 33–64) minutes on scene assessing and, if necessary, treating the patient. After assessment, 617 patients (40.9%) were reassigned or handed over to a different prehospital unit (e.g. an ambulance or supine transportation without a need for treatment), while treatment was completed on scene for a considerable proportion of the patients (29.1%), both without conference with a physician (12.1%), in consultation with the EMCC physician (12.1%) and after conference with the MECU physician (4.9%).

**Table 2:** Characteristics of all dispatches and patients served by the PVU (*n* = 1.510)

Characteristic	
Sex, *N (%)*	
Male	701 (46.4%)
Female	631 (41.8%)
Missing^a^	178 (11.8%)
Age group, *N (%)*	
Under 18	87 (5.8%)
18–34	263 (17.4%)
35–49	169 (11.2%)
50–64	252 (16.7%)
65–79	315 (20.9%)
80+	244 (16.2%)
Missing^a^	180 (11.9%)
Level of urgency, *N (%)*	
A	132 (8.7%)
B	1263 (83.6%)
Other	115 (7.6%)
Time to arrival (minutes), *median (IQR)*	14 (10–19)^b^
On scene time (minutes), *median (IQR)*	49 (33–64)
End of treatment, *N (%)*	
Patient reassigned to or handed over to other prehospital	617 (40.9%)
Treatment completed on site without consultation with a physician	183 (12.1%)
Treatment completed on site by consultation with EMCC-physician	182 (12.1%)
Referred to own transport	134 (8.9%)
Referred to seated transport	76 (5.0%)
Treatment completed on site by consultation with MECU-physician	74 (4.9%)
System cancellation or cancelled while driving	57 (3.8%)
Patient refused transport	49 (3.3%)
Operational consideration	42 (2.8%)
Incident not relevant	21 (1.4%)
Patient not present on site	11 (0.7%)
Missing	64 (4.3%)

^a^Some patients served by the PVU did not have a personal identification number noted in their prehospital medical record, hence their age and sex is unknown

^b^Excluded: 8 observations with registration errors (e.g. arrival time of 0 min)

**Table 3:** Main presenting problem among patients served by the PVU (*n* = 1.510)

Main presenting problem	*N* (%)	Age^a^
Impaired consciousness, paralysis, and dizziness	241 (16.2%)	67 (48–79)
Unclear problem	212 (14.3%)	62 (33–78)
Breathing problems	172 (11.6%)	71 (56–81)
Abdominal and back pain	154 (10.4%)	51 (51–67)
Accidents (not traffic-related)	147 (9.9%)	67 (36–80)
Seizures	130 (8.8%)	25 (14–48)
Chest pain, heart disease	108 (7.3%)	52 (32–73)
Alcohol, poisoning and overdose	79 (5.3%)	51 (37–62)
Psychiatry, suicidal	40 (2.7%)	30 (23–56)
Minor wound, fracture, injury	34 (2.3%)	73 (46–79)
Bleeding, non-traumatic	27 (1.8%)	76 (60–83)
Unconscious	23 (1.6%)	73 (60–82)
Traffic accident	16 (1.1%)	31 (25–57)
Diabetes	14 (1.0%)	46 (35–75)
Other	88 (5.8%)	60 (33–77)
Missing	25 (2.0%)	67 (54–73)

^a^Median (IQR)

In the interviews, four themes were identified that were represented broadly among all professions: (1) The implementation strategy had gaps, but was supported by ongoing adjustments, (2) Facilitating a patient-centered approach for the benefit of the patient and the system, (3) Community partnership and internal collaboration enabled paramedics as healthcare facilitators, and (4) Flexible workflows were needed to maintain professional agency. The themes along with descriptions and additional quotes are presented in Table 4.

**Table 4:** Overview of results of the thematic analysis with explanatory quotes (*n* = 19)

Theme	Content	Explanatory quotes
The Implementation Strategy Had Gaps, But Was Supported by Ongoing Adjustments	Lack of timely information about the implementation process caused frustration. However, there was great willingness from the management to implement ongoing changes as issues were raised.	- Paramedic 2: “Things get introduced without us being fully informed. Just all of a sudden. But they also said in the interview: ‘We’ll be paving the road as we drive.’ And that’s fine enough. But I would perhaps like it if those of us who are actually on the ground were involved in the process from the beginning, because after all, it’s us who have to do the work.” - Paramedic 1: “This project group has really listened to the things I mentioned during the interview. And they had the right people involved, so in that sense, I think the leadership has been good. I like that part”.
Facilitating a Patient-Centered Approach for the Benefit of the Patient and the System	The PVU was justified by the professionals by: (1) optimal use of scarce resources within the healthcare system, and (2) patient-centered and high-quality patient care delivered by the PVU.	- Paramedic 1: “So, I think, from a socio-economic perspective, it’s the best thing I’ve ever been a part of”. Interviewer: It’s the PVU?”. Paramedic 1: “Yes, because 37%, who don’t undergo hospitalization at costs of 25,000 DKK each, well. It speaks for itself. I need just one patient, one patient to justify my own salary. Then I need one patient to stay home every 5 days. Then I’ve earned my own salary.” - Technical dispatcher 7: “I think it’s a good idea, and it makes sense from a holistic perspective. But, I could easily use an extra ambulance.”
Community Partnership and Internal Collaboration Enables Paramedics as Healthcare Facilitators	Internal collaboration within the EMS as well as partnerships across the broader healthcare system were perceived as important for effective and high-quality operation of the PVU.	- EMS dispatcher 5: “We have a really good collaboration with the PVU. They often come up and talk to us about tasks, so we discuss a lot. We receive a lot of professional consultation, and I learn a great deal from them. Almost every time they are on duty, they come up for a cup of coffee and talk about the tasks they’ve been on, and… so, I think, in terms of both professional consultation and the collegial aspect, we have a really good collaboration.” - Technical dispatcher 3: “I think it’s both that we trust each other, but also because if I say to him, ‘I’m super under pressure,’ he does it a bit faster than if it’s someone I don’t have a relationship with because then he knows that I only say: ‘I’m under pressure’, when I really am.”
Flexible Workflows Were Needed to Maintain Professional Agency	A common challenge was lack of agency and professional recognition, which restricted their work, due to guidelines restricting the PVU’s operation.	- Paramedic 2: “We should also move away from the fact that I have to confer with the EMCC physician. I would like it to be the case that we COULD call, as it is right now, we MUST call the EMCC physician. And if it were the case that you could call if you were in doubt and consult with the EMCC physician, because it’s a good idea to have an EMCC physician as a backup. But it’s intimidating that you HAVE to with every patient. That you can’t assess it yourself. Not that I can’t go out and assess this professionally, but that I have to ask my ‘dad’ or ‘mom’ for permission first…” - EMS dispatcher 5: “But in situations where we can say with 100% certainty, ‘I know she needs to go in.’ Then, I think it would be nice to be able to choose not to send it *[the PVU]* ourselves. Because then we’re in a situation where some resources could be better used elsewhere.”

### Theme 1: the implementation strategy had gaps, but was supported by ongoing adjustments

As a new addition to the existing prehospital set-up, the implementation of the PVU naturally surfaced as a key theme throughout the interviews. Certain aspects of the implementation process were experienced as lacking. The main issue was related to the timing and lack of information; some felt that the amount of information provided was insufficient, and information often arrived late. EMS dispatchers mentioned lacking information in proper time about which patients should be triaged to the PVU, while paramedics operating the unit needed more time adjusting to new ways of working, e.g. getting to know the new vehicle and learning to work alone, as illustrated in the following quote:

*We didn’t know what to do. What was the task*,* what can we do? Here’s a car. GO. That’s how it felt. We start now*,* ‘okay’; They also admit that from the management. It came together quickly. – Paramedic 5*.

As a result, the professionals lacked a clear understanding of the rationale behind the new initiative before it was implemented. However, several interviewees mentioned a great willingness from the management to make ongoing changes as problems became apparent. Notably, concerns raised in early interviews were absent in later ones, highlighting the gradual adjustments in response to staff feedback. An example of management’s responsiveness is illustrated in the following quote by one of the EMS dispatchers:

*I think it’s been good that they started in a specific area*,* not the entire region*,* avoiding chaos everywhere. They tried it on a small scale initially and made adjustments before*,* hopefully*,* expanding to more locations. I also appreciate that they have been receptive when we’ve come up with ideas like*,* “Could this unit be used for these patients too?” They listened to it and adjusted the index [the Danish Index for Emergency Care]*,* so it aligns well now. …*.*– EMS dispatcher 6*

### Theme 2: facilitating a patient-Centered approach for the benefit of the patient and the system

In general, there was a pattern of positive attitudes towards the PVU; the professionals recognized its purpose and found the work meaningful. The PVU was acknowledged as benefitting both the patients and the healthcare system. Most professionals believed that the PVU saved resources, especially within emergency departments, as the PVU contributed to the prevention of hospital admissions. Moreover, the professionals recognized a great potential for more patients being treated and discharged on scene. This is illustrated below:

*At least in my time in the emergency department*,* I experienced that a lot of patients arrived who*,* in essence*,* could have either been treated at home or could have avoided hospitalization*, *if there had just been some ‘healthcare eyes’ on the scene. – EMS dispatcher 2*.

Furthermore, the professionals felt that the PVU delivered better and more patient-centered care, especially in cases of doubt or difficulties with triaging the patient, where the PVU provided ‘eyes’ on scene. The paramedics had sufficient time to find the best solution for the patient, contrary to working on the ambulance, where they were more conscious about being available for the next assignment, thus seeing transportation to the hospital as an easy solution. Also, on scene treatment was perceived as the best and most considerate option for the patient, when deemed possible and safe. Although some patients may still need conveyance to the hospital, the work of the PVU was not considered wasted resources, as the transportation might be handled by other means than an ambulance, and diagnostics and treatment could be initiated on scene by the paramedics. However, the technical dispatchers had a somewhat different perspective. They did recognize the purpose of the PVU, but the ambulances were understaffed, which complicated their work. Therefore, the majority of technical dispatchers would prefer to cut the PVU, if it meant having more ambulances in service.

### Theme 3: community partnership and internal collaboration enabled paramedics as healthcare facilitators

A key theme that was identified across interviews was collaboration, defined broadly as both internal collaboration within the EMS and external partnerships with the broader healthcare system. Good and effective collaboration was highlighted as a necessity for the PVU to be viable and deliver quality patient care. For paramedics, working collaboratively with external healthcare professionals was an essential part of their new role operating the non-conveyance solo-ambulance. Although the paramedics were physically alone on scene, they had vast resources available to them through phone calls, e.g. they had the possibility of contacting the EMCC and have a different EMS vehicle dispatched to the patient, when deemed necessary. Furthermore, they could contact various forms of social services and specialized hospital departments to find the right level of care for the patient. One paramedic even referred to his phone as the most important tool in providing the best solution for the patient. The paramedics were placed in a new role, where they had to navigate the healthcare system on behalf of the patient, which could only be done through good collaboration, as illustrated in the quote below:

*No*,* we can’t solve the tasks alone. For example*,* this patient. I need to talk to the hospital*,* I need to talk to a patient-transport car*,* I am not alone. – Paramedic 3*.

*You are just pulling the strings? - Interviewer*

*Yes, I am facilitating a healthcare system. (…) And the thing about conveying a message. In this case I have to disseminate a healthcare system and be an ambassador for the patient. – Paramedic 3*

Internal teamwork between the paramedic, the EMS dispatcher and the technical dispatcher represented a different aspect of collaboration, necessary for the effective dispatch and operation of the PVU. In this context, professional consultation, trust, and clear communication between the different professions were emphasized as essential for ensuring the PVU operating effectively, ultimately resulting in improved patient care. For instance, the EMS dispatchers asked the technical despatchers about the availability of the PVU before triaging a patient to it. Furthermore, the paramedics shared their experiences of operating the PVU with the EMS dispatchers at the EMCC, thereby provided learning opportunities for the healthcare professionals triaging the patients.

### Theme 4: flexible workflows were needed to maintain professional agency

A common challenge across professions, although expressed in different ways, was the lack of agency and professional recognition, which impeded the professionals’ autonomy in performing tasks that they deemed most appropriate for the patient and the operation of the PVU. Paramedics expressed frustration about lacking agency and recognition of their professional skills and having to “ask for permission”, when completing patient treatment on-scene by conferring with a physician. In many cases, paramedics felt they could safely make the decision to discharge the patient on scene without consulting a physician. However, guidelines stated that consultation with either the EMCC physician or the physician in the MECU was mandatory for certain symptoms or patient groups.

Likewise, regulations hindered the EMS dispatchers and the technical dispatchers in deploying the PVU to some patients due to guidelines restricting the use of the PVU. Several of the dispatchers found the triage instructions inflexible and initially confusing. Adhering to these instructions caused frustration at the EMCC, especially in highly acute and life-threatening situations, where the PVU was the closest vehicle to the patient, but they were not allowed to dispatch it according to the guidelines. These frustrations led to bending rules to potentially save lives, as illustrated in the below quote:

*I think it’s something like children with febrile seizures*,* it’s not supposed to be sent*,* but if they’re right next to it*,* and the ambulance has a 10-minute drive*,* then I’m not supposed to send it*,* but I do it anyway*,* so I have to stand by that decision*,* and I will always do that*,* and if they don’t think I should have done it*,* then they can terminate me. It’s a bit black and white… – Technical dispatcher 2*.

## Discussion

This mixed-methods study had a convergent parallel design that combined quantitative and qualitative data to comprehensively examine characteristics of patients assessed by the PVU during the first year of operation, how patients were managed within the EMS, when triaged to the PVU, as well as how the healthcare professionals partaking in the dispatch and operation of the PVU perceived their expanded role and responsibilities. We found that the PVU provided patient-centered care to a broad range of patients, especially in situations assessed as urgent, but not life-threatening, where paramedics acted as a facilitator for the broader healthcare system on behalf on the patient. The findings highlight the importance of strong collaboration, as the paramedics take on this new role working as a single responder without the option of transporting patients to the hospital. Furthermore, the results emphasize the importance of responsive management and flexible workflows in implementing such initiatives to support and preserve professionals’ sense of agency.

In many cases, the paramedics operating the PVU were able to complete treatment on scene, contributing towards reducing avoidable hospital admissions. However, 40.9% of patients attended to by the PVU were handed over to a different prehospital unit after assessment by the paramedic, which raises the question of whether the PVU was just adding another layer to the patients’ pathway through the healthcare system. This concern was echoed in the interviews by some of the dispatchers, when the PVU was initially implemented. On the other hand, these numbers could indicate that the PVU acts as a facilitator to other healthcare resources after face-to-face assessment that cannot be provided through telephone triage. This was reflected in the qualitative results, where paramedics perceived themselves as navigating the healthcare system on behalf of the patients, collaborating with other healthcare stakeholders (e.g. general practitioners, the EMCC physician, hospital departments) to determine and access the right level of care for patients. This is in line with previous research on non-conveyance, highlighting the importance of collaboration with various professionals to provide optimal patient care, including the presence of alternative care options for low-acuity patients, which provides a safety-net for patients after non-conveyance [30–32].

A patient group frequently attended to by the PVU were those with non-specific complaints; 14.3% of patients treated during the first year of operation called the EMS with an unclear problem, while 16.2% of patients presented with symptoms classified as “impaired consciousness”. Both categories represent a variety of states and conditions, making it difficult to determine the appropriate level of care without further assessment. Non-specific complaints are especially common among older patients, and as the aging population continues to grow, the number of patients presenting with such complaints is increasing [33, 34]. The PVU appears to offer a viable alternative within the EMS for these patients, where hospital admission may not be necessary, yet a more detailed assessment on scene is required to ensure appropriate care and decision-making. This was perceived as valuable by the professionals, particularly when triage of patients was difficult. Hence, the PVU provided “healthcare eyes” on scene and clarification for patients, while the paramedics had ample time to thoroughly assess the patient, with a median time of 49 (IQR: 33–64) minutes spent on scene for evaluation and treatment, if needed.

Across the professions interviewed, various challenges were encountered throughout the implementation process, mainly due to lacking information and inflexible guidelines restricting the use of the PVU. This inner struggle between following non-conveyance guidelines and doing what is perceived as best for the patients has previously been described by ambulance clinicians in relation to non-conveyance encounters [29]. In the present study, the healthcare professionals called for more agency and respect for their professional competency when making decisions about the dispatch and operation of the PVU, especially when they recognized a potential for its expanded use, e.g. for highly acute or life-threatening situations. Throughout its first year of operation, the PVU was dispatched to 132 patients (8.7%) assigned the highest level of urgency, reflecting the changes made throughout the implementation process, as the scope of the PVU was expanded to include assignments with urgency level A, when the PVU was the nearest vehicle to highly acute and life-threatening situations. Such ongoing adjustments are crucial for successful implementation, as implementation processes should be flexible, non-linear, and responsive to key stakeholders [22]. Healthcare professionals, in particular, play a vital role in this success, as the real implementation occurs in every interaction between patient and professional [35]. In the current study, PVU guidelines were refined throughout its first year of operation to better align with the perspectives of the professionals dispatching and operating the PVU. Effective implementation requires such adaptation to the context in which it occurs [22].

### Strengths and limitations

We selected a mixed-methods study design, combining qualitative and quantitative data, which allowed for a deeper and more nuanced understanding of the patient care provided by the PVU than any of the data sources could have provided on their own. However, it could be considered a limitation of the study that we were able to “fact check” any statements made by the professionals in the interviews by looking at the quantitative data on which assignments the PVU had carried out throughout the data collection period. Therefore, one could argue that the perspectives and experiences of the professionals were not allowed to speak for themselves. However, the qualitative and quantitative data were analyzed by different researchers with no access to the other data or results, which should mitigate any unwarranted “fact checking”.

Previous studies have mainly investigated the perspectives of ambulance personnel, e.g. ambulance nurses [29, 30, 32], while the perspectives of employees triaging patients to and dispatching these units have been overlooked. Hence, a key strength of the current study is that we were able to combine perspectives from all professions involved in the dispatch and operation of the PVU. As the professions work closely together, triangulating their perspectives was important for gaining a comprehensive understanding.

The researchers conducting the interviews did not share the technical background of the informants, which could have limited potential bias in the interview process. Without prior assumptions, the researchers were less likely to introduce or reinforce existing presumptions or take the professionals’ experiences for granted. However, they did carry out participant observation prior to the interviews to gain knowledge and be able to engage meaningfully with the professionals. Along with our inductive approach applied during the data analysis, this allowed us to explore new and unexpected perspectives on the PVU that could not have been anticipated.

The selection of participants could have introduced bias, as participants were chosen by scheduling coordinators within the EMS and not sampled by the research group. Therefore, selection was limited by the schedules and availability of staff, which could have biased the results. However, at the same time, this approach ensured that only employees with solid experience working with the PVU were recruited for the study, as the extend of experience of the employees was known by the scheduling coordinators.

While generalizability of the findings across different settings is limited when programs are tailored to specific contexts, it also highlights the strength of the PVU, as community paramedic programs should always be responsive to local needs [14, 36]. Caution should be exercised when applying these findings to other settings, as the current study was performed in one region only. Hence, important aspects of the context enabling successful implementation might differ in other regions and countries, e.g. non-conveyance guidelines, response times and possibilities of the ambulance clinicians to refer and collaborate with other parts of the healthcare system. Also, comparison with existing literature was constrained by the sparseness of knowledge currently available on similar non-conveyance initiatives, especially focusing on employee perspectives.

## Conclusions

This study adds to the sparse literature on novel non-conveyance initiatives, which can be applied within other EMS systems planning to implement similar initiatives. The PVU was perceived as offering patient-centered care while contributing to efficient use of healthcare resources. The PVU seems to offer a valuable alternative within the EMS, particularly for patients with non-specific complaints and conditions that can be managed at a lower level of care than hospitalization. As prehospital EMS increasingly respond to non-acute medical needs, alternative EMS vehicles like the PVU can play an important role in meeting future demands. However, for successful implementation, ongoing adjustments are required, particularly in maintaining the professional autonomy of employees operating and dispatching the vehicle.

## Supplementary Information

Additional file 1: Interview guide – Technical dispatchers.

Additional file 2: Interview guide – EMS dispatchers.

Additional file 3: Interview guide – Paramedics.

Additional file 4.

## Data Availability

The data has not been made available on a permanent third-party archive given the nature of the data and the small number of participants, as well as General Data Protection Regulations (GDPR). However, access to the data (in anonymized form) will be granted from the corresponding author upon request but may require the completion of a formal data sharing agreement, in compliance with GDPR. The interview guide is available as additional files 1-3.

## Abbreviations

EMS: Emergency Medical Services
PVU: Prehospital Visitation Unit
IQR: Interquartile range
EMCC: Emergency Medical Coordination Center
MECU: Mobile Emergency Care Unit
POCT: Point-of-care testing
CRP: C-reactive protein
