# Supplementary material for: Is non-conveyance solo-ambulances a useful mean to meet the increasing demand for emergency medical services in Denmark?
Source: BMC Health Serv Res. 2025 Feb 25;25:307. doi: 10.1186/s12913-025-12448-8 (PMC11852878; doi:10.1186/s12913-025-12448-8)
Supplement: Supplementary file 1 — Additional file 1: Interview guide – Technical dispatchers. [file 12913_2025_12448_MOESM1_ESM.docx]

***Supplementary File 2: Interview guide – EMS dispatchers***

| **Research question** | | **Interview question** |  |
| --- | --- | --- | --- |
| Briefing | | |  |
| **Introduction** | ***Introduction of the interviewer and research project***    ***Walk-through of the consent form*** | |  |
| **Workflows** | | |  |
| Work experience | | Could you briefly describe your professional background and how many years you have worked as a technical dispatcher? |  |
| Investigate the impact of the PVU on the workflow of the EMS dispatchers | | What is the PVU?    What do you see as the purpose of the PVU?    What impact has the introduction of the PVU had on your workflow? |  |
| **Working environment** | | |  |
| Investigate the impact of the PVU on the working environment of the EMS dispatchers | | How has it been referring patients to a different type of service than you normally do? |  |
|  |  | How has it been with the introduction of a new unit in the prehospital care that, among other things, plays a referral role at the scene of an incident?    Do you see the PVU as helpful for the overall task of prehospital care? How? |  |
|  |  | Has the implementation of the PVU changed your view on the professional competencies of the paramedics? How so?    Have you gained a greater insight into what a paramedic can do? How so? |  |
| **The implementation process** | | |  |
| Investigate the EMS dispatchers’ experience with the implementation process | | How were you informed about the implementation of the PVU?  How was the amount of information provided during the initial phase of the PVU? |  |
|  |  | What were your thoughts on the PVU, when you first heard about it? |  |
|  |  | How was the implementation carried out? |  |
|  |  | What has been handled correctly in connection with the implementation of the PVU?    Is there anything you would like to be done differently? What/why? |  |
|  |  | Were you well-prepared to dispatch the PVU from the start? If not, what did you feel you were missing? |  |
|  |  | What was the atmosphere like in the department during the implementation of the PVU? |  |
|  |  | How was the internal collaboration within the prehospital EMS during the implementation of the PVU? |  |
|  | | Now that it's been almost a year, how would you describe the implementation process?*    Do you feel that, as an EMS dispatcher, you are part of an ongoing evaluation of the PVU? * |  |
| **The future of the PVU** | | |  |
| Investigate the EMS dispatchers’ perspective on future potential and purposes of the PVU | | Do you think the PVU can be used for purposes other than what it was intended for?    What do you see as the ideal scenario for the use of the PVU? |  |
|  |  | Do you see a need for any changes regarding the PVU? |  |
|  |  | What benefits and drawbacks do you see in having the PVU as a new and additional vehicle to dispatch? |  |
|  |  | Which advantages does the PVU have compared to the ambulances? |  |
|  |  | The goal in the project description for the implementation of the PVU is, among other things, to ensure better referral of patients when it is unclear what the patient’s condition is – have you experienced that the PVU has fulfilled this goal? |  |
| **Debriefing** | | |  |
|  | | Would you like to add anything else?    ***Thank the informant for participating*** |  |
|  |  |  |  |

*Question added for the last interview round.
