# Supplementary material for: Is non-conveyance solo-ambulances a useful mean to meet the increasing demand for emergency medical services in Denmark?
Source: BMC Health Serv Res. 2025 Feb 25;25:307. doi: 10.1186/s12913-025-12448-8 (PMC11852878; doi:10.1186/s12913-025-12448-8)
Supplement: Supplementary file 2 — Additional file 2: Interview guide – EMS dispatchers. [file 12913_2025_12448_MOESM2_ESM.docx]

**Online Supplementary Material**

***Supplementary File 1: Interview guide – Technical dispatchers***

| **Research question** | | **Interview question** |
| --- | --- | --- |
| Briefing | | |
| **Introduction** | ***Introduction of the interviewer and research project***    ***Walk-through of the consent form*** | |
| **Workflows** | | |
| Work experience | | Could you briefly describe your professional background and how many years you have worked as a technical dispatcher? |
| Investigate the impact of the PVU on the workflows of the technical dispatchers | | How does dispatching of a prehospital response work after receiving a referral from the EMS dispatcher?    What is the PVU?  What is the purpose of the PVU? |
|  |  | What changes has the PVU made to your workflows?    When does the PVU come into play for you during a workday? |
| **Working environment** | | |
| Investigate the impact of the PVU on the working environment and workload for the technical dispatchers | | How has it been to dispatch an additional vehicle with different dispatching options?    How does it work only being able to dispatch the PVU in specific areas – and disposing over it therefore changes from day to day? |
|  |  | Have you experienced situations where the PVU is the closest vehicle to the incident site, but it is not possible to dispatch it to the scene?   - Have you experienced any changes?*     Can you provide any examples? How does it feel being in such situations? |
|  |  | How is the collaboration with the PVU paramedics? |
| **The implementation process** | | |
| Investigate the technical dispatchers’ experience with the implementation process | | How were you informed about the implementation of the PVU?    How was the amount of information provided during the initial phase of the PVU? |
|  |  | What were your thoughts on the PVU, when you first heard about it? |
|  |  | How was the implementation carried out? |
|  |  | What has been handled correctly in connection with the implementation of the PVU?    Is there anything you would like to be done differently? What/why? |
|  |  | Were you well-prepared to dispatch the PVU from the start? If not, what did you feel you were missing? |
|  |  | What was the atmosphere like in the department during the implementation of the PVU? |
|  |  | How was the internal collaboration within the prehospital EMS during the implementation of the PVU? |
|  | | Now that it's been almost a year, how would you describe the implementation process? *    Do you feel that, as a technical dispatcher, you are part of an ongoing evaluation of the PVU?* |
| **The future of the PVU** | | |
| Investigate the technical dispatchers’ perspective on future potential and purposes of the PVU | | Do you think the PVU can be used for purposes other than what it was intended for?    What do you see as the ideal scenario for the use of the PVU? |
|  |  | What changes do you think are needed regarding the PVU? |
|  |  | Do you see a purpose for the PVU in other areas of the Central Denmark Region? |
|  |  | Do you see a purpose for the PVU at other times during the day or week? |
|  |  | What benefits and drawbacks do you see in having the PVU as a new and additional vehicle to dispatch? |
|  |  | Having to choose between the PVU and an additional ambulance, which would you choose and why? |
|  |  | How much would you expect the PVU to be used before it proves beneficial? |
| **Debriefing** | | |
|  | | Would you like to add anything else?    ***Thank the informant for participating*** |
|  |  |  |

*Question added for the last interview round.
