# Supplementary material for: Is non-conveyance solo-ambulances a useful mean to meet the increasing demand for emergency medical services in Denmark?
Source: BMC Health Serv Res. 2025 Feb 25;25:307. doi: 10.1186/s12913-025-12448-8 (PMC11852878; doi:10.1186/s12913-025-12448-8)
Supplement: Supplementary file 3 — Additional file 3: Interview guide – Paramedics. [file 12913_2025_12448_MOESM3_ESM.docx]

***Supplementary File 3: Interview guide – Paramedics***

| **Research question** | **Interview question** |
| --- | --- |
| Briefing | |
| **Introduction** | ***Introduction of the interviewer and research project***    ***Walk-through of the consent form*** |
| **Workflows and Point Of Care Testing (POCT)** | |
| Work experience | Could you briefly describe your professional background and how many years you have worked as a paramedic?    What was your motivation for being part of the PVU team?    Where do you operate, when driving the ambulance? |
| Investigate the impact of the PVU on the workflows of the paramedics | What is the PVU?    What do you see as the purpose of the PVU? |
|  | Can you describe a typical workday operating the PVU? |
|  | What have you experienced the PVU being used for?    What patients are the PVU typically dispatched to? |
|  | What do you do when you are unsure about the treatment of a patient while operating the PVU?    What do you do when you are unsure about the referral or discharge of a patient while operating the PVU? |
|  | Which equipment does the PVU carry that the ambulance does not? |
|  | Do you think it makes sense to have a lactate meter in the PVU? Why or why not?    Do you use it? |
|  | How do your professional competencies match the needs of the PVU?    Do you experience professional growth by working independently in the PVU? – In what ways? |
|  | Have you experienced that working on the PVU has changed your mindset compared to operating the ambulance?    How has it been not being able to transport the patient?  Are you more focused on discharging the patient on scene, when operating the ambulance? |
|  | How can the experiences from the PVU be applied to other emergency services? |
|  | How have the other paramedics responded to the PVU?     - Have you exchanged experiences with colleagues – *who do not operate the PVU* – regarding completing treatment and discharging the patient on scene? |
| **Working environment** | |
| Investigate the impact of the PVU on the working environment of the paramedics | How has it been splitting your work time between the PVU and the ambulance, spending half of your time on each? |
|  | How do you feel about operating the PVU? |
|  | What are your thoughts on continuing to work with the PVU after the 6-month trial period is over?    *(The PVU often deals with a different patient type, typically chronic patients who are more complex, while paramedics are trained for acute situations, which the PVU does not handle to the same extent)* |
|  | How has it been working alone with the patient? |
| **Collaborating partners** | |
| Investigate the paramedics’ experience of collaborating partners’ reactions regarding the PVU | Who are the partners you collaborate with, when operating the PVU? |
|  | What do you experience the partners (in-hospital departments, municipal emergency teams, geriatric units etc.) is saying about the establishment of the PVU? |
|  | How have you experienced working with the emergency department of the Aarhus university hospital? |
|  | How have you experienced working with the regional hospital of Gødstrup? |
|  | How have you experienced working with the EMCC? |
| **Patients and their relatives** | |
| Investigate the paramedics’ experience of patients and their reactions on the PVU | What type of patient do you most often refer to other units if you are unable to discharge them at the scene?    What do you most often refer them to?    Do you find that you are often able to discharge patients at home? |
|  | What reactions have the patients had being attended to by the PVU?  How do the patients react when they realize it’s the PVU that has arrived, meaning they cannot be transported to the hospital?  How do the patient's relatives react? |
|  | How do you perceive the patient safety of the PVU? Why do you consider it safe or not safe for the patient? |
| **The implementation process** | |
| Investigate the paramedics’ experience with the implementation process | How were you informed about the implementation of the PVU?    How was the amount of information provided during the initial phase of the PVU? |
|  | What were your thoughts on the PVU, when you first heard about it? |
|  | How was the implementation carried out? |
|  | What has been handled correctly in connection with the implementation of the PVU?    Is there anything you would like to be done differently? What/why? |
|  | I understand that you completed a one-day educational course before operating the PVU. Do you feel that you were well-prepared to operate the PVU from the start?    Is there anything you felt was missing in the course? |
|  | What was the atmosphere like in the department during the implementation of the PVU? |
|  | How was the internal collaboration within the prehospital EMS during the implementation of the PVU? |
| **The future of the PVU** | |
| Investigate the EMS dispatchers’ perspective on future potential and purposes of the PVU | Do you think the PVU can be used for purposes other than what it was intended for?    What do you see as the ideal scenario for the use of the PVU? |
|  | Do you think the PVU is a good solution to the resource challenges in the Gødstrup area? Why/why not? |
|  | Do you see a purpose for the PVU in other areas of the Central Denmark Region? |
|  | Do you see a purpose for the PVU at other times during the day or week? |
|  | Does the PVU have additional capabilities, such as completing treatment on scene, that could be beneficial if expanded for use in the ambulances? |
|  | What advantages does the PVU have compared to ambulances? What about disadvantages? |
|  | How much would you expect the PVU to be used before it proves beneficial? |
|  | What changes do you think are needed? |
|  | The goal in the project description for the implementation of the PVU is to prevent unnecessary hospital admissions, ensure better referral of patients when their condition is unclear, and ensure referral to the appropriate mode of transport. Have you experienced that the PVU has met these goals? |
| **Debriefing** | |
|  | Would you like to add anything else?    ***Thank the informant for participating*** |
