# Supplementary material for: Is non-conveyance solo-ambulances a useful mean to meet the increasing demand for emergency medical services in Denmark?
Source: BMC Health Serv Res. 2025 Feb 25;25:307. doi: 10.1186/s12913-025-12448-8 (PMC11852878; doi:10.1186/s12913-025-12448-8)
Supplement: Supplementary file 4 — Additional file 4. [file 12913_2025_12448_MOESM4_ESM.docx]

| **Guideline** | **Section: page** |
| --- | --- |
| Describe the justification for using a mixed methods approach to the research question | Methods, study design: page 2-3 |
| Describe the design in terms of the purpose, priority and sequence of methods | Methods, study design: page 2-3 |
| Describe each method in terms of sampling, data collection and analysis | Methods, data collection: page 5-7  Methods, data analysis: page 7-8 |
| Describe where integration has occurred, how it has occurred and who has participated in it | Methods, study design: page 2 |
| Describe any limitation of one method associated with the present of the other method | Discussion, Strengths and limitations: page 19 |
| Describe any insights gained from mixing or integrating methods | Discussion: page 17-19 |

**Additional File 4: Good Reporting of A Mixed Methods Study’ (GRAMMS) checklist**
